# Statin Use and Primary Open-Angle Glaucoma Severity in Patients of African Ancestry

**DOI:** 10.21203/rs.3.rs-9911540/v1

**Published:** 2026-06-11

**Authors:** Joan O’Brien, Marine-Ayan Ibrahim Aibo, Isabel Di Rosa, Laxmi Moksha, Fangming Jin, Aaron Zhao, Yan Zhu, Rebecca Salowe, Roy Lee, Victoria Addis, Gui-shuang Ying

**Affiliations:** Penn Medicine; Penn Medicine; University of Pennsylvania Perelman School of Medicine; Scheie Eye Institute; University of Pennsylvania Perelman School of Medicine; University of Pennsylvania; University of Pennsylvania

**Keywords:** primary open-angle glaucoma (POAG), African ancestry, statins

## Abstract

**Objective:**

The evidence supporting the benefit of statins in the treatment of primary open-angle glaucoma (POAG) is mixed, and their effect among patients of African ancestry is largely unexplored. This study examines the association of statins with the prevalence and severity of POAG in this ancestry group.

**Design:**

Cross-sectional study.

**Subjects:**

5715 subjects of African ancestry (African American, African descent, or African Caribbean) ages 35 and older, recruited from ophthalmology clinics in the University of Pennsylvania Health System as part of the Primary Open-Angle African Ancestry Glaucoma Genetics (POAAGG) study.

**Methods:**

Logistic regression was used to analyze the prevalence of POAG in statin users versus non-users, adjusting for demographic and clinical co-variates. Sub-analyses compared POAG prevalence between high-potency and low-potency statin users. Ocular endophenotypes were compared between statin users and non-users.

**Main Outcome Measures:**

POAG prevalence, ocular endophenotypes.

**Results:**

POAG prevalence did not differ between statin users and non-users (OR: 0.99, 95% CI: 0.85, 1.14; p = 0.84). Among POAG cases, statin users had significantly smaller mean cup-to-disc ratio (mean difference [MD]=−0.03, 95% CI: −0.04, −0.01, p < 0.001), better visual acuity (MD=−0.10, 95% CI: −0.16, −0.04, p = 0.001), thicker mean retinal nerve fiber layers (MD = 1.43, 95% CI: 0.18, 2.68, p = 0.03), and lower mean pattern standard deviation values (MD=−0.39, 95% CI: −0.66, −0.13, p = 0.004) than non-users.

**Conclusion:**

Statin use was not associated with POAG prevalence but was associated with milder structural and functional disease features among cases. These findings suggest the need for further investigation of the effect of statins in African ancestry patients with POAG.

## Introduction

Glaucoma is the leading cause of irreversible blindness worldwide.(1) Primary open-angle glaucoma (POAG), the most common form of the disease, has an estimated global population prevalence of 2.4%, affecting over 68 million individuals.(1),(2) POAG disproportionately affects Black patients, who are estimated to be up to four times more likely to have POAG than white patients.(2),(3),(4) This disease is characterized by slow, progressive optic nerve damage with loss of retinal ganglion cells (RGC) and corresponding loss of visual field (VF).(5) Because early disease is often asymptomatic, up to half of affected patients are unaware they have the disease, suggesting the worldwide disease burden may be substantially underestimated.(3) Glaucomatous vision loss can result in considerable functional impairment for patients, including reduced mobility and an increased frequency of falls, problems with facial recognition, and hindrance to tasks crucial for independent living, such as reading and driving.(6, 7)

The complete pathogenesis of POAG is not fully understood and the only modifiable clinical target remains the reduction of intraocular pressure (IOP), modified through medications, laser, or surgical intervention.^1^ Although elevated IOP is the most common risk factor for the development of POAG, some patients, including an estimated 14–57% of Black patients with POAG, suffer from normal-tension glaucoma (NTG), in which optic nerve damage progresses despite normal IOP levels.(9),(10),(11, 12) This highlights the need for additional treatment avenues beyond targeting IOP reduction.

Statins, inhibitors of HMG-CoA reductase widely prescribed for hyperlipidemia and cardiovascular disease prevention, have drawn attention in recent years for the potential treatment of glaucoma. Statins have been proposed to influence glaucoma-related pathways through modulation of Rho/ROCK signaling and potential neuroprotective effects.(13, 14) Statins have a well-established safety profile, and their use is widespread. However, despite their benefit and common clinical indications, they are less likely to be prescribed to and used by Black patients.(15, 16) Studies have shown that statins may provide benefit in the treatment of POAG both through direct neuroprotective effects and through the mediation of aqueous humor outflow, although the evidence is mixed – some studies find benefit, while others find increased risk of glaucoma in statin users.(17) Recent work has revealed that these disparate results may be mediated by differential effects by racial and ethnic groups.(18) The use of statins for protection against the development of POAG in a solely African ancestry cohort has yet to be studied.(19–23)

In this paper, we investigate the association of statin use on POAG prevalence and severity in African ancestry patients. The Primary Open-Angle African Ancestry Glaucoma Genetics (POAAGG) study, a large glaucoma genetics study in Philadelphia, aims to investigate the genetic factors and characteristics associated with POAG in patients who self-identify as Black. The POAAGG study includes 10,255 subjects of African ancestry and has led to key understandings about the manifestations of POAG in this ancestry group, including the identification of several likely causal genetic variants that could ultimately be used to help guide treatment.(4) Leveraging this large, well-characterized cohort, we aimed to clarify whether statins are associated with the development of POAG and with ocular structural and functional measures of disease severity.

## Methods

### Study Population

Subjects in this study were recruited from the larger cohort of the POAAGG study. POAAGG subjects were recruited from July 2010 to July 2019, self-identified as Black (African American, African descent, or African Caribbean), and were aged ≥ 35 years.(24) Subjects were recruited during regularly scheduled visits to the Ophthalmology Department at the University of Pennsylvania, as well as during visits to several nearby ophthalmology clinics and at outreach events in Philadelphia. At enrollment, subjects received an onsite interview as well as a comprehensive ophthalmic evaluation. A glaucoma specialist or ophthalmologist classified each subject as a POAG case, suspect, or control. Subjects were identified as POAG cases if they had: (1) an open anterior chamber angle, (2) characteristic glaucomatous findings in one or both eyes upon optic nerve examination, (3) characteristic VF deficits in one or both eyes consistent with observed optic nerve defects on two consecutive tests, and if (4) secondary causes of glaucoma were excluded.(24) Subjects were identified as controls without POAG if they were 35 or older and did not have the following: high myopia, high presbyopia, abnormal VF, IOP above 21 mmHg, abnormal optic nerve characteristics, asymmetry, or cup-to-disc ratio (CDR) difference between two eyes greater than 0.2.(24) A DNA sample from blood or saliva was collected for genomic analysis from POAAGG subjects. Details on POAAGG study eligibility, patient demographics, data collection, and phenotyping have been previously published.(24, 25) All subjects provided informed consent, and the study was approved by the Institutional Review Board at the University of Pennsylvania. This study was conducted according to the guidelines of the Declaration of Helsinki.

For this particular study, inclusion criteria included enrollment at a University of Pennsylvania Health System (UPHS) site and classification as a POAG case or control. These criteria allowed use of electronic medical records (EMR) to be evaluated for statin use. Subjects with a history of rho-kinase inhibitor (netarsudil/Rhopressa) prescription were excluded because a proposed mechanism of statins’ impact on glaucoma involves the Rho/ROCK pathway. A total of 5 715 subjects were included in this study.

### Medication history

Patient charts from UPHS were searched for current statin, rho-kinase inhibitor, and systemic beta-blocker prescriptions. Subjects were labeled as statin users if they had a prescription for a statin, and as statin non-users if they did not have an active statin prescription. Statins or statin-containing drugs included in search terms were atorvastatin (Lipitor, Atorvaliq), atorvastatin-amlodipine (Caduet), ezetimibe-simvastatin (Vytorin), fluvastatin (Lescol), lovastatin (Mevacor, Altoprev), pitavastatin (Livalo, Zypitamag), pravastatin (Pravachol), rosuvastatin (Crestor, Ezallor Sprinkle), and simvastatin (Zocor, FloLipid). Statin users were labeled as high-potency users if prescribed a high-potency statin (atorvastatin or rosuvastatin) or were labeled as low-potency users if prescribed any other statin. Systemic beta-blocker use was determined and controlled for given the known IOP-lowering effect of beta-blockers, which has been a significant confounder in prior studies examining statins’ effect on POAG.(26) Subjects with a prescription for a Rho-kinase inhibitor at the time of this study (Rhopressa/netarsudil) were excluded to minimize confounding effects.

### Demographic characteristics

Demographic characteristics collected at enrollment included age, sex, body mass index (BMI), history of diabetes mellitus, history of hypertension, family history of glaucoma, history of previous glaucoma surgery, age at glaucoma diagnosis, beta blocker prescription, alcohol use, and tobacco use. All variables were supplemented through EMR, as described previously.(24)

### Ocular endophenotypes

Ocular endophenotypes were obtained during the onsite examination or within 6 months of POAAGG enrollment and were stored in Research Electronic Data Capture.(27, 28) As subjects returned for subsequent visits, endophenotypes were updated in the POAAGG database. These phenotypes were measured and analyzed at the eye level and included IOP, CDR, visual acuity (VA), VF mean deviation (MD) and pattern standard deviation (PSD), and retinal nerve fiber layer (RNFL) thickness.

### Statistical Analysis

Statistical analysis was conducted to compare the prevalence of POAG by count and percentage in statin users and non-users. This was first conducted using an unadjusted logistic regression, then with a logistic regression adjusted for age, sex, BMI, diabetes mellitus history, hypertension history, family glaucoma history, history of previous glaucoma surgery, beta-blocker use, tobacco use, and alcohol use. Sub-analyses were performed comparing statin non-users to high-potency statin users and low-potency statin users.

For demographic characteristics, categorical variables were analyzed using count and percentage and compared with chi-square tests. Odds ratios (ORs) were derived by Wald Confidence Interval (CI). The mean difference (95% CI) and p-value of continuous variables were compared with t-tests. The alpha for all statistical tests was set at α = 0.05.

Ocular endophenotypes values were analyzed at the eye level, and p-values and difference (95% CI) were calculated based on a linear regression model, adjusting for inter-eye correlation with generalized estimating equations (GEE).

## Results

A total of 5715 subjects and 11 430 eyes were included in this study. Of those subjects, 3302 (6 604 eyes) were controls and 2413 (4826 eyes) were POAG cases.

In statin users versus non-users, we found no statistical difference between the prevalence of POAG (OR = 0.99; 95% CI: 0.85, 1.14; p = 0.84) based on a logistic regression model adjusted for age, sex, BMI, diabetes mellitus history, hypertension history, family glaucoma history, history of previous glaucoma surgery, beta-blocker use, tobacco use, and alcohol use (Supplemental Table 1). The distribution of statin users, including by high- and low-potency statins, and non-users in cases and controls is described in Table 1. Of controls, 48.85% used high-potency statins, 10.81% used low-potency statins, and 40.34% did not use statins. Of cases, 41.15% used high-potency statins, 16.08% used low-potency statins, and 42.77% did not use statins. When comparing the statin user sub-groups, neither high-potency statin users (prevalence 38.1%, OR = 0.95; 95% CI: 0.81, 1.10; p = 0.5) nor low potency users (prevalence 52.1%, OR = 1.13; 95% CI: 0.91, 1.40; p = 0.28) differed significantly in prevalence of POAG from non-users (Supplemental Table 1).

Among POAG cases, statin users were significantly younger (mean difference [MD]=−1.38; 95% CI: −2.33, −0.42; p = 0.005) and had a higher mean BMI (MD = 1.64; 95% CI: 1.12, 2.17; p < 0.001), higher risk of diabetes mellitus (OR = 2.29; 95% CI: 1.93, 2.72; p < 0.001), and higher risk of hypertension (OR = 2.00; 95% CI: 1.64, 2.43; p < 0.001) than statin non-users (Table 2). Statin users were also more likely to be prescribed a systemic beta blocker (OR = 2.18; 95% CI: 1.82, 2.60; p < 0.001), have history of tobacco use (OR = 1.25; 95% CI: 1.06, 1.47; p = 0.008), and were less likely to have previous glaucoma surgery (OR = 0.70; 95% CI: 0.59, 0.84; p < 0.001) than statin non-users (Table 2).

Table 3 describes ocular phenotypes in POAG cases, comparing statin users and non-users. Among POAG cases, statin users had slightly lower mean CDR (MD=−0.03; 95% CI: −0.04, −0.01; p < 0.001), better visual acuity (MD=−0.10 (95% CI: −0.16, −0.04), p = 0.001), lower PSD (MD=−0.39; 95% CI: −0.66, −0.13; p = 0.004), and thicker RNFL (MD = 1.43; 95% CI: 0.18, 2.68; p = 0.03) compared to non-users. There was no difference in mean IOP or VF MD.

## Discussion

This study examined the association between statin use and POAG prevalence and severity in subjects of African ancestry. While statin use was not significantly associated with POAG prevalence, several ocular endophenotypes differed significantly between POAG cases using and not using statins, especially related to optic nerve health. Cases using statins had lower CDR and thicker mean RNFL, suggesting healthier optic nerve structure, as well as better VA and lower PSD than non-users. Notably, mean IOP did not differ between groups, consistent with existing literature reporting no association between statin use and IOP in POAG.(22) These findings suggest that statins may confer a benefit to optic nerve health and visual function in POAG independent of IOP reduction.

Prior studies have shown mixed evidence regarding the impact of statin use on various measures related to POAG. Although some studies have shown a null association or higher risk of glaucoma with statin use, others suggest a decreased risk of POAG in individuals using statins.(29),(30, 31) Elhusseiny et al. recently found that these differential findings may be due to differing effects of statins on POAG in patients of varying ethnic backgrounds, identifying that statin use in non-Hispanic African American patients was associated with a reduction in open-angle glaucoma risk at one and five years.(18)

The proposed protective effect of statins likely involves multiple pathways. N-methyl-D-aspartate (NMDA) receptors, expressed throughout the retina, have been linked to RGC death due to excessive glutamatergic input. This mechanism is hypothesized to play a role in the pathogenesis of glaucoma, and *in vitro* analyses have shown that atorvastatin attenuates glutamate-induced excitotoxicity independent of HMG Co-A reductase inhibition.(32, 33) Statins have also been found to reduce RGC apoptosis and glial activation in a rat model of chronic ocular hypertension.(14) Another critically important potential mechanism through which statins may be protective in glaucoma involves the Rho/Rho-associated coiled-coil protein kinase pathway (ROCK). Statins inhibit the Rho/Rho-kinase (ROCK) pathway in atherosclerotic disease, and the Rho-kinase pathway has been implicated in optic nerve damage in glaucoma over the last two decades.(13),(34) Topical Rho-kinase inhibitors, such as netarsudil, are now available as a novel and effective therapy for glaucoma.(34, 35) Of particular importance to patients of African ancestry, a large genome-wide association study (GWAS) recently published by our group identified variants in *ROCK1P1*, a pseudogene of ROCK1, and *ARHGEF12*, whose product functions downstream of the RhoA/Rho-associated kinase pathway, as two likely causal variants for POAG in this patient population.(4) The ability of statins to inhibit the Rho/ROCK pathway in cardiovascular diseases independent of their cholesterol-lowering properties suggests that a similar protective mechanism could be at play in POAG.(13)

There were several limitations to this study. The absence of a significant association with disease prevalence may relate to the cross-sectional design and the lack of data on treatment duration. Our inclusion criteria captured subjects prescribed statins at the time of data analysis, but did not account for treatment duration or adherence. It is possible that some subjects had been on statins for an insufficient period to exert a protective effect. Several prior studies have reported differential effects of short term (< 2 years) and long-term (> 2 years) statin exposure on POAG risk.(20–22),(29) Future research should stratify statin use by treatment duration to clarify the long-term impact of statins on POAG in individuals of African ancestry. Another limitation was presented by the variables controlled for in this study. For instance, non-statin cholesterol-lowering agents have also been found to independently lower the risk of OAG, but were not controlled for.(20) Although we adjusted and controlled for known confounders such as beta-blocker and rho kinase inhibitor use, and age, residual confounding remains possible, particularly from non-statin cholesterol-lowering agents.(20, 36) Finally, given the observational nature of this study and stated limitations, causal inference cannot be established.

Nonetheless, our large, well-characterized cohort and detailed endophenotypic data strengthen our findings and underscore the importance of further clinical and mechanistic studies investigating statins as potential neuroprotective therapies for POAG in this high-risk population.

## Supplementary Material

Supplementary Files

This is a list of supplementary files associated with this preprint. Click to download.

• Table1StatinsEyeSubmission.xlsx

• Table2StatinsEyeSubmission.xlsx

• Table3StatinsEyeSubmission.xlsx

• SupplementalTable1StatinsEyeSubmission.xlsx

## Abbreviations

POAG: primary open-angle glaucoma
RGC: retinal ganglion cells
POAAGG: Primary Open-Angle African Ancestry Glaucoma Genetics
IOP: study, intraocular pressure
NTG: normal-tension glaucoma
HMG CoA Reductase: 3-hydroxy-3-methylglutaryl-CoA reductase
ROCK: Rho-associated, coiled-coil-containing protein kinase
GWAS: genome-wide association study
UPHS: University of Pennsylvania Health System
BMI: body mass index
EMR: electronic medical records
CDR: cup-to-disc ratio
VA: visual acuity
VF: visual field
MD: VF mean deviation
PSD: pattern standard deviation
RNFL: retinal nerve fiber layer
OD: right eye
OS: left eye
OR: odds ratio
CI: Wald Confidence Interval
GEE: generalized estimating equations
NMDA: N-methyl-D-aspartate receptors
